# Prompt Engineering as an Important Emerging Skill for Medical Professionals: Tutorial

**DOI:** 10.2196/50638

**Published:** 2023-10-04

**Authors:** Bertalan Meskó

**Affiliations:** 1 The Medical Futurist Institute Budapest Hungary

**Keywords:** artificial intelligence, AI, digital health, future, technology, ChatGPT, GPT-4, large language models, language model, LLM, prompt, prompts, prompt engineering, AI tool, engineering, healthcare professional, decision-making, LLMs, chatbot, chatbots, conversational agent, conversational agents, NLP, natural language processing

## Abstract

Prompt engineering is a relatively new field of research that refers to the practice of designing, refining, and implementing prompts or instructions that guide the output of large language models (LLMs) to help in various tasks. With the emergence of LLMs, the most popular one being ChatGPT that has attracted the attention of over a 100 million users in only 2 months, artificial intelligence (AI), especially generative AI, has become accessible for the masses. This is an unprecedented paradigm shift not only because of the use of AI becoming more widespread but also due to the possible implications of LLMs in health care. As more patients and medical professionals use AI-based tools, LLMs being the most popular representatives of that group, it seems inevitable to address the challenge to improve this skill. This paper summarizes the current state of research about prompt engineering and, at the same time, aims at providing practical recommendations for the wide range of health care professionals to improve their interactions with LLMs.

## The Emergence of Large Language Models and Prompt Engineering

With the emergence of large language models (LLMs), with the most popular one being ChatGPT that has attracted the attention of over a 100 million users in only 2 months, artificial intelligence (AI), especially generative AI has become accessible for the masses [1]. This is an unprecedented paradigm shift not only because of the use of AI becoming more widespread but also due to the possible implications of LLMs in health care [2].

Numerous studies have shown what medical tasks and health care processes LLMs can contribute to in order to ease the burden on medical professionals, increase efficiency, and decrease costs [3].

Health care institutions have started investing in generative AI, medical companies have started integrating LLMs into their businesses, medical associations have released guidelines about the use of these models, and medical curricula have also started covering this novel technology [4-6]. Thus, a new, essential skill has emerged: prompt engineering.

Prompt engineering is a relatively new field of research that refers to the practice of designing, refining, and implementing prompts or instructions that guide the output of LLMs to help in various tasks. It is essentially the practice of effectively interacting with AI systems to optimize their benefits.

In the context of medical professionals and health care in general, this could encompass the following:

- Decision support: medical professionals can use prompt engineering to optimize AI systems to aid in decision-making processes, such as diagnosis, treatment selection, or risk assessment.
- Administrative assistance: prompts can be engineered to facilitate administrative tasks, such as patient scheduling, record keeping, or billing, thereby increasing efficiency.
- Patient engagement: prompt engineering can be used to improve communication between health care providers and patients. For example, AI systems can be designed to send prompts for medication reminders, appointment scheduling, or lifestyle advice.
- Research and development: in research scenarios, prompts can be crafted to assist in tasks such as literature reviews, data analysis, and generating hypotheses.
- Training and education: prompts can be engineered to facilitate the education of medical professionals, including ongoing training in the latest treatments and procedures.
- Public health: on a larger scale, prompt engineering can assist in public health initiatives by helping analyze population health data, predict disease trends, or educate the public.

Prompt engineering, therefore, has the potential to improve the efficiency, accuracy, and effectiveness of health care delivery, making it an increasingly important skill for medical professionals.

This paper summarizes the current state of research on prompt engineering and, at the same time, aims at providing practical recommendations for the wide range of health care professionals to improve their interactions with LLMs.

## The State of Prompt Engineering

The use of LLMs, especially ChatGPT, comes with major limitations and risks. First, since ChatGPT is not updated in real time and its training data only include information up to November 2021, it may lack crucial, up-to-date medical research or changes in clinical guidelines, potentially impacting the quality and relevance of its responses. Furthermore, ChatGPT cannot access or process individual user data or context, which limits its ability to provide personalized medical advice and increases the risk of data misinterpretation.

There is also a crucial need for users to verify every single response from ChatGPT with a qualified health care professional, as the model's answers are generated on the basis of patterns in the data it was trained on and may not be accurate or safe.

The model's inability to empathize or deliver sensitive information may also result in a subpar patient experience. Importantly, potential breaches of patient confidentiality could violate privacy laws such as the Health Insurance Portability and Accountability Act of 1996 in the United States. Despite its potential as an assistive tool, these limitations necessitate careful consideration of its application in health care [7].

While these risks are significant, the potential outcomes can outweigh them; therefore, the need for improving at designing better prompts has grown extensively since the launch of ChatGPT.

There have been attempts at addressing this issue. One study aimed at designing a catalogue of prompt engineering techniques, presented in pattern form, which have been applied to solve common problems when conversing with LLMs [8]. Another study provided a summary of the latest advances in prompt engineering for a very specific audience, researchers working in natural language processing for the medical domain, or academic writers [9,10]. One study introduced the potential of an AI system to generate health awareness messages through prompt engineering [11].

While there is research in the field, it is clear that there have been no comprehensive, yet practical guides for medical professionals. This is the gap that this paper aims to fill.

## How to Improve at Prompt Engineering

As in the case of any essential skill, becoming better at prompt engineering would involve an improved understanding of the fundamental principles of the technology, gaining practical exposure to systems using the technology, and continually refining and iterating the skill based on feedback.

The following are some concrete steps that a health care professional can take to improve their skills in prompt engineering:

- Understanding the underlying principles of how AI and machine learning models work can provide a foundation on which to build prompt engineering skills. As shown, it is possible to gain that understanding without any prior technical or coding knowledge [12].
- Familiarizing themselves with the LLMs they are working with as each system has its own set of capabilities and limitations. Understanding both can help craft more effective prompts.
- Practice makes perfect; therefore, attempting to interact with LLMs regularly and make a note of the prompts that yield the most helpful and accurate results can have benefits.

It is also important to constantly test prompts in real-world scenarios as their effectiveness is best evaluated in practical application.

## Specific Recommendations for Better LLM Prompts

**Figure 1:**
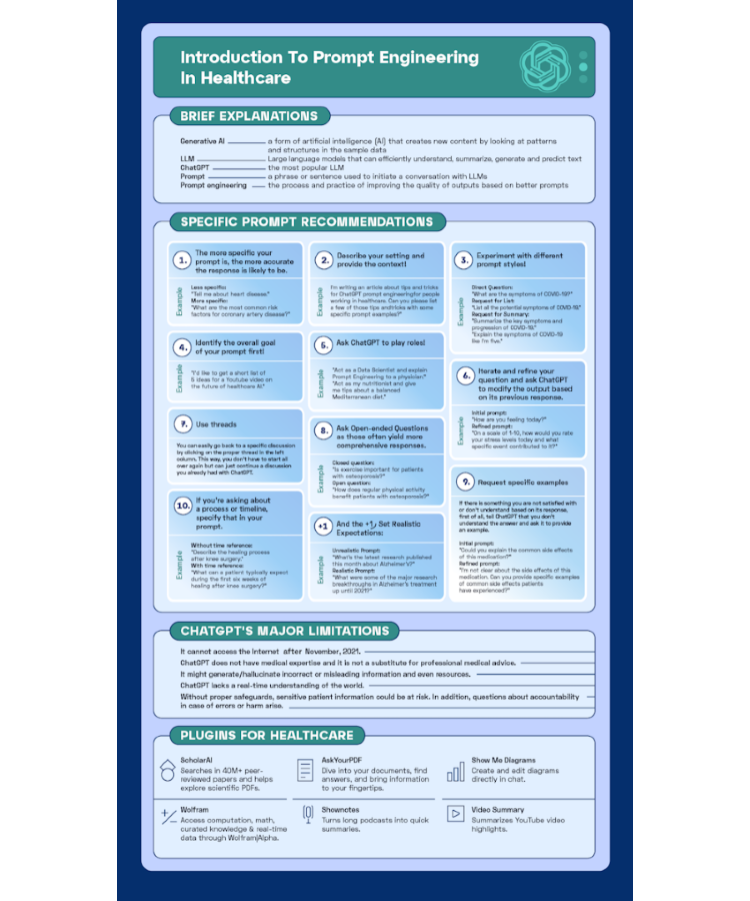
A cheat sheet of prompt engineering recommendations for health care professionals with examples for each: ChatGPT’s key terms and their explanations, its limitations, and its most popular plugins. A high resolution version is attached as Multimedia Appendix 1.

Besides these general approaches, here is a summary of specific recommendations with practical examples that a health care professional might want to consider to improve their skills in prompt engineering. Figure 1 summarizes these recommendations, their examples with ChatGPT’s key terms, limitations, and the most popular plugins.

### Be as Specific as Possible

The more specific the prompt, the more accurate and focused the response is likely to be. The following is an example prompt:

- Less specific: “Tell me about heart disease.”
- More specific: “What are the most common risk factors for coronary artery disease?”

### Describe the Setting and Provide the Context Around the Question

One must consider the discussion one is having with ChatGPT as a discussion one would have with a person they just met, who might still be able to answer their questions and address one’s challenges.

The following is an example prompt: “I'm writing an article about tips and tricks for ChatGPT prompt engineering for people working in healthcare. Can you please list a few of those tips and tricks with some specific prompt examples?”

### Experiment With Different Prompt Styles

The style of one’s prompt can significantly impact the answer. One can try different formats such as asking ChatGPT to generate a list about their brief or to provide a summary of the topic. The following is an example:

- Direct question: “What are the symptoms of COVID-19?”
- Request for a list: “List all the potential symptoms of COVID-19.”
- Request for a summary: “Summarize the key symptoms and progression of COVID-19.”
- Process: “Provide a step-by-step process of diagnosing COVID-19.”

### Identify the Overall Goal of the Prompt First

Describe exactly what kind of output is being sought. Whether it would be getting creative ideas for an article, asking for a specific description of an advanced scientific topic, or providing a list of examples around questions, defining it helps ChatGPT come up with more relevant answers. The following is an example: “I'd like to get a list of 5 ideas for a presentation at a scientific event to make my research findings more easily understandable.”

### Ask it to Play Roles

This can help streamline the desired process of obtaining the information or input one was looking for in a specific setting. With new topics without prior knowledge, it is prudent to obtain only a basic description; in addition, one can also ask ChatGPT to act as a tutor and help dive into a detailed topic step-by-step. The following are a couple of examples:

- “Act as a Data Scientist and explain Prompt Engineering to a physician.”
- “Act as my nutritionist and give me tips about a balanced Mediterranean diet.”

### Iterate and Refine

Even if one’s skills in prompt engineering are advanced, LLMs change so dynamically that one rarely get the best response on was looking for after the first prompt attempt. Constantly iterating prompts is something with which we should get accustomed. Users of LLMs are also encouraged to ask the LLM to modify the output based on feedback on its previous response.

### Use the Threads

One can navigate back to a specific discussion by clicking on the specific thread in the left column on ChatGPT’s dashboard. This way, one can build upon the details and responses one has already received in a previous thread. This can save a lot of time as there is no need to describe the same situation and all the feedback ChatGPT has received on its responses.

### Ask Open-Ended Questions

Open-ended questions can provide a broader, more comprehensive understanding of the user's situation. For instance, asking “How do you feel?” rather than “Do you feel pain?” allows for a wider array of responses that can potentially provide more insight into the patient's mental, emotional, or physical state. Open-ended questions can also help to generate a larger data set for training AI models, making them more effective. Lastly, asking open-ended questions allows ChatGPT to display its potential better by leveraging its training on a diverse range of topics. This can lead to more unexpected and creative solutions or ideas that a health care professional might not have thought of. The following is an example:

- Closed question: “Is exercise important for patients with osteoporosis?”
- Open question: “How does regular physical activity benefit patients with osteoporosis?”

### Request Examples

Asking for specific examples can help to clarify the meaning of a concept or idea, making it easier to understand. Especially with complex medical terminology or procedures, examples can provide a practical context that aids comprehension. Also, examples often help in visualizing abstract or complicated ideas. When ChatGPT provides examples, it can showcase how a certain concept or rule is applied in different scenarios. This can be beneficial in health care, where theoretical knowledge needs to be connected to real-world applications.

### Temporal Awareness

This refers to the model's understanding of time-related concepts and its ability to generate contextually relevant responses based on time. Therefore, describing what time line the prompt and the desired output would be related to helps LLMs provide a more useful answer. The following is an example:

- Without a time reference: “Describe the healing process after knee surgery.”
- With a time reference: “What can a patient typically expect during the first six weeks of healing after knee surgery?”

### Set Realistic Expectations

Knowing the limitations of AI tools such as ChatGPT is crucial, as it helps set realistic expectations about the output. For instance, ChatGPT cannot access any data or information after November, 2021; it cannot provide personalized medical advice or replace a professional's judgement. The following is an example:

- Unrealistic prompt: “What's the latest research published this month about Alzheimer's?”
- Realistic prompt: “What were some of the major research breakthroughs in Alzheimer's treatment up until 2021?”

### Use the One-Shot/Few-Shot Prompting Method

The one-shot prompting method is one in which ChatGPT can generate an answer based on a single example or piece of context provided by the user. The following is an example:

- Generate 10 possible names for a new digital stethoscope device.
- A name that I like is DigSteth.

With the few-shot strategy, ChatGPT can generate an answer based on a few examples or pieces of context provided by the user. The following is an example:

- Generate 10 possible names for a new digital stethoscope device.
- Names that I like include:
  Digital
  Steth
  Stethoscope

### Prompting for Prompts

One of the easiest ways of improving at prompt engineering is asking ChatGPT to get involved in the process and design prompts for the user. The following is an example: “What prompt could I use right now to get a better output from you in this thread/task?”

## Conclusions

As the skill of prompt engineering has gained significant interest worldwide, especially in the health care setting, it would be important to include teaching the practical methods this paper described in the medical curriculum and postgraduate education. While the technical details and background of generative AI will probably be included in future curricula, it would be useful for medical students to learn the most practical tips of using LLMs even before that happens.

The general message for every LLM user should be that they could use such AI tools to expand their knowledge, capabilities, and ideas instead of solving things on their behalf. Ideally, this approach and mindset would stem from trained medical professionals who could share it with their patients.

In summary, as more patients and medical professionals use AI-based tools—LLMs being the most popular representatives of that group—it seems inevitable to address the challenge to improve at this skill. Furthermore, as doing so does not require any technical knowledge or prior programming expertise, prompt engineering alone can be considered an essential emerging skill that helps leverage the full potential of AI in medicine and health care.

## Appendix

Multimedia Appendix 1 Higher resolution version of Figure 1.

## Abbreviations

AI: artificial intelligence
LLM: large language model
