# Supplementary figures and images for: Prompt Engineering as an Important Emerging Skill for Medical Professionals: Tutorial
Source: J Med Internet Res. 2023 Oct 4;25:e50638. doi: 10.2196/50638 (PMC10585440; doi:10.2196/50638)

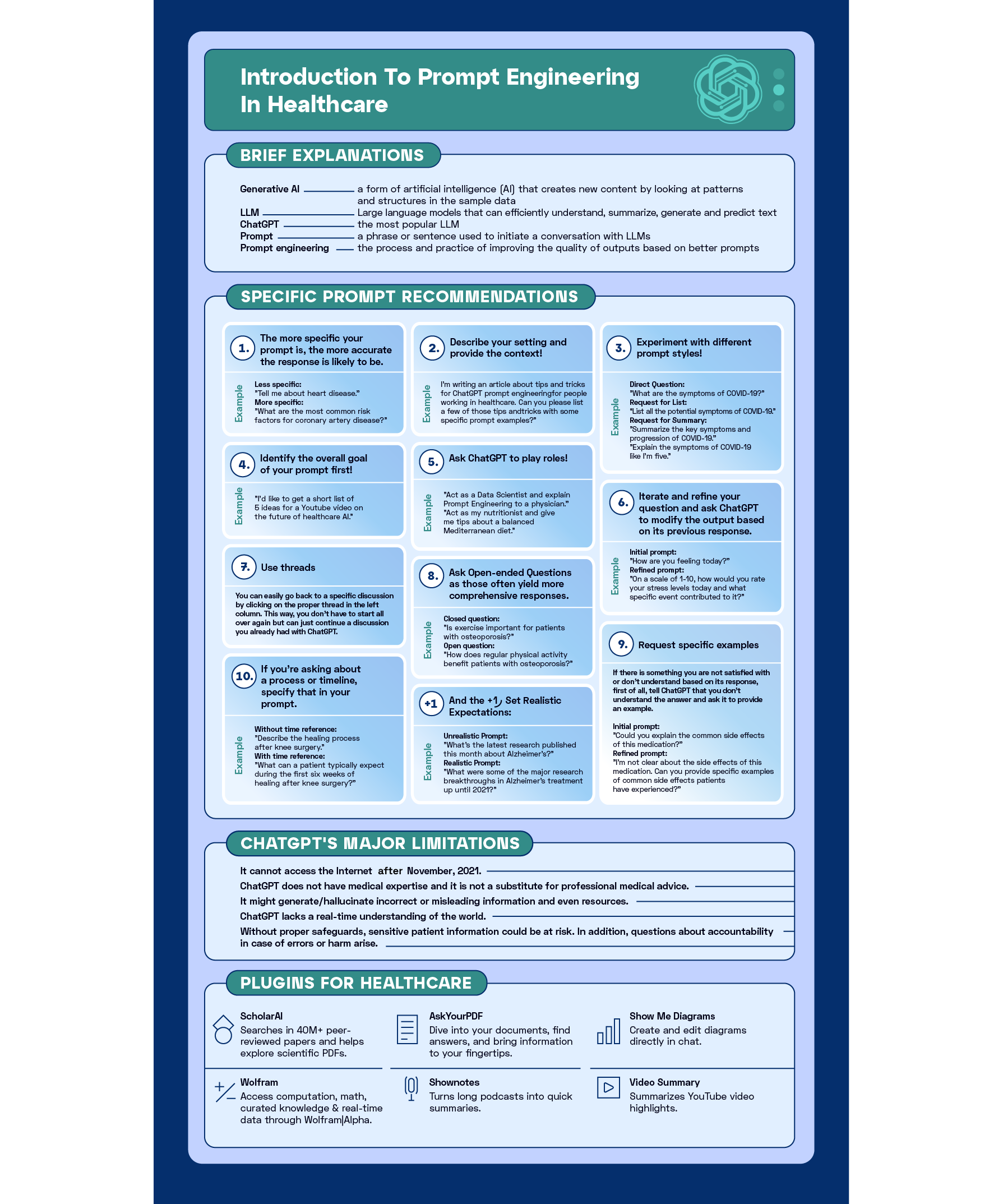

Supplement: Multimedia Appendix 1 [file jmir_v25i1e50638_app1.png]
